# Monozygotic dichorionic diamniotic twins with large interstitial deletion of chromosome 1p

**DOI:** 10.1002/ccr3.2358

**Published:** 2019-08-06

**Authors:** Leah Yieh, Christina Ramo, Cori Feist, Brian L. Shaffer, Amanda Kim

**Affiliations:** ^1^ Division of Neonatology, Department of Pediatrics, Keck School of Medicine Children's Hospital Los Angeles, Fetal and Neonatal Institute, University of Southern California Los Angeles CA USA; ^2^ Department of Pediatrics Oregon Health & Science University Portland OR USA; ^3^ Department of Obstetrics and Gynecology Oregon Health & Science University Portland OR USA

**Keywords:** chromosome 1p, interstitial deletion, monozygotic twins

## Abstract

We describe twins with an interstitial deletion of chromosome 1 with a severe phenotype compared to previously described cases. As genetic testing is more frequently performed, it is important for clinicians to understand the spectrum of clinical findings that can occur with this particular deletion.

## 1 INTRODUCTION

We describe the clinical course of the first case of monozygotic twins prenatally diagnosed with a rare deletion of the short arm of chromosome 1 (46,XX,del(1)(p22p32)). The patients had more severe phenotypic features than previously reported cases. We highlight the striking differences in potential phenotypes for interstitial deletions involving the short arm of chromosome 1, ranging from mild cognitive impairment to more severe phenotypic abnormalities as seen in these twins, including microretrognathia, congenital heart defects, and suspected vasculopathy predisposing them to necrotizing enterocolitis. As prenatal genetic testing becomes more readily available, this information can aid clinicians in identifying similar cases and facilitate counseling.

De novo interstitial deletions of the short arm of chromosome 1 are rare and have been infrequently described in the literature. Chromosome 1 is the largest human chromosome and contains approximately 2000 known genes, making up 8% of the human genome.^1^ The majority of rearrangements of chromosome 1 involve the long arm. Terminal deletions of the short arm are more common though a few cases of interstitial deletions have been reported in the literature.^2^, ^3^, ^4^, ^5^ Most of the published reports were identified in older children or adults in the setting of developmental delay and minor structural anomalies.^2^, ^3^, ^6^, ^7^, ^8^, ^9^, ^10^ In this paper, we present monozygotic twins with a large interstitial deletion of chromosome 1p with more severe phenotypic features than previously described.

## 2 CLINICAL REPORT

The patients were born to a 19‐year‐old primigravid mother who had irregular prenatal care. Family history was negative for congenital anomalies, cognitive impairment, multiple spontaneous abortions, stillbirths, consanguinity, or suspected syndromic conditions. Pregnancy was notable for a dichorionic diamniotic twin gestation, tobacco abuse, and substance use (methamphetamines in the first trimester and tetrahydrocannabinol in third trimester). Remainder of routine prenatal laboratories was unremarkable. Serial fetal ultrasounds in the 2nd trimester showed many structural anomalies in both fetuses. Common findings in each twin included cystic hygroma, single umbilical artery, strawberry‐shaped skull, cerebral ventriculomegaly, retromicrognathia, probable cardiac defect, pericardial effusion, and clenched hands. Both twins had normal appearing female external genitalia. The lower extremities of twin A were noted to have an overlapping appearance, whereas twin B was noted to have a left‐sided choroid plexus cyst, suspected absence of the right kidney, and echogenic bowel. Cell‐free DNA (cf DNA) screening was low risk for trisomy 13, 18, and 21 and consistent with XX sex chromosomes. Fetal echocardiogram revealed multiple ventricular septal defects (VSDs) in twin A and evidence of cardiac diastolic dysfunction in twin B. Amniocentesis revealed a large interstitial deletion of chromosome 1p22‐p32 by karyotype of cultured cells (46,XX,del(1)(p22p32)) from both twins. Single nucleotide polymorphism microarray was performed and confirmed a 31.71MB deletion of 1p32.3‐p22.2 encompassing dozens of OMIM genes (1p32.3‐p22.2 57652246_89311711). Parental karyotypes were normal, suggesting de novo deletion or gonadal mosaicism.

The mother was referred to our fetal care program to discuss prognosis and make a plan for the delivery. She presented at 32 weeks gestation where ultrasound confirmed the previous findings. In addition, twin B had segmentation anomalies of the sacrum, areas of delayed brain sulcation, absent cavum septum pellucidum, and suspected severe dysgenesis or agenesis of the corpus callosum with resultant colpocephaly. Twin A had a structurally normal brain by ultrasound. Upon presentation to our clinic, the mother was painfully contracting. Fetal heart rate monitoring for twin A was notable for minimal variability and late decelerations, so she was transferred to the labor and delivery unit where preterm rupture of membranes occurred. Due to concern for fetal acidemia in the setting of progressing preterm labor, the twins were delivered via cesarean due to transverse lie of twin A.

**Figure 1:**
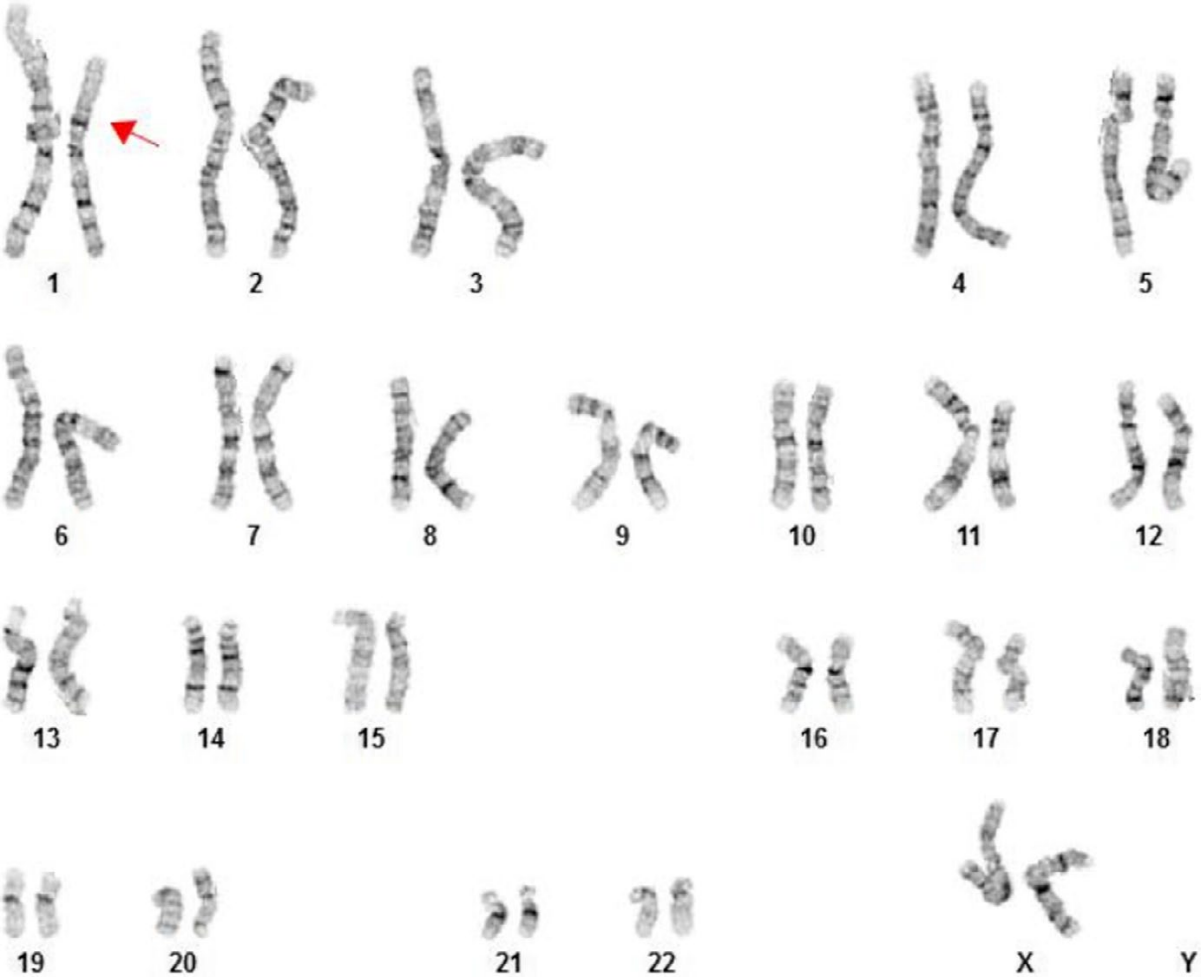
Giemsa‐banded karyogram of Twin A demonstrating 46,XX,del(1)(p32.2p22.2)

**Figure 2:**
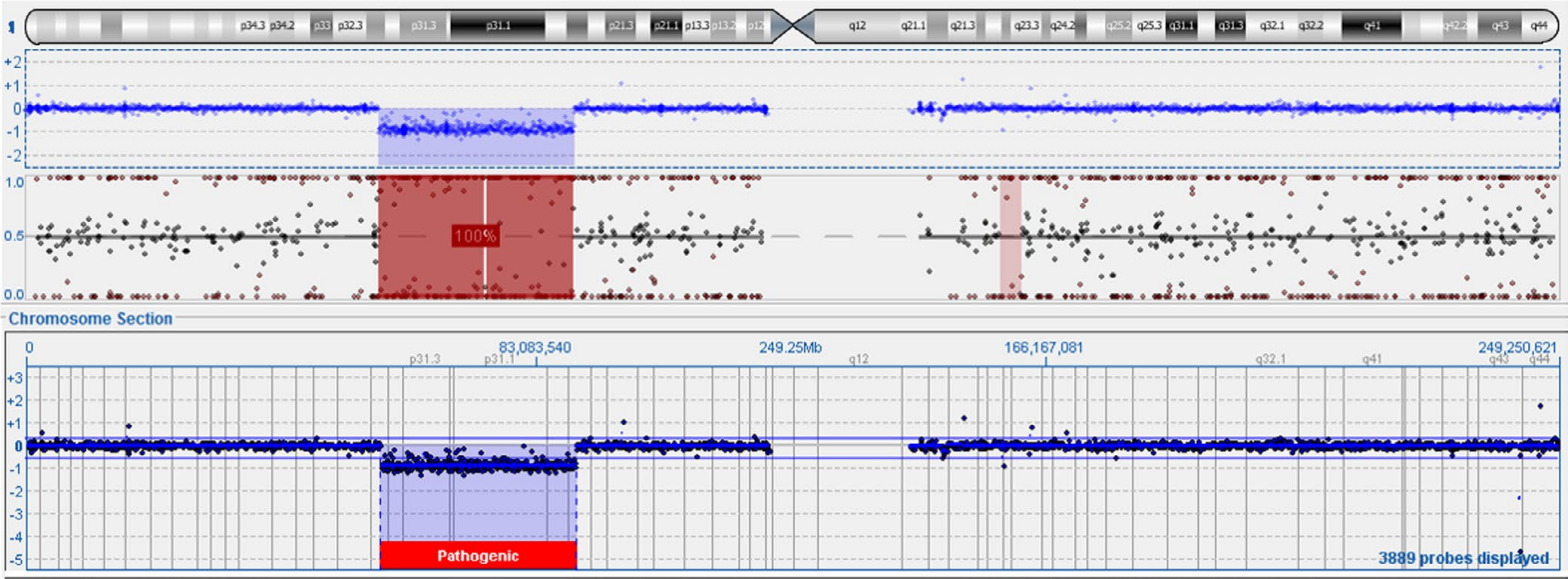
Microarray of Twin A demonstrating arr[19] 1p32.2p22.2(57652246_89311771)x1. Upper track shows single nucleotide polymorphisms with absence of heterozygosity due to deletion. Lower track shows oligonucleotide probes indicating deletion

The prenatally diagnosed 31.71Mb de novo deletion of chromosome 1p22.2‐32.2 was confirmed postnatally on umbilical cord blood from both twins (Figures 1 and 2). Due to similar anomalies and identical deletions yet dichorionicity by ultrasound and placental pathology, zygosity testing was performed and consistent with monozygous twins.

A summary of each subject's anomalies is provided in Table 1. Here, we will highlight the hospital course by system.

**Table 1:** Comparison of clinical phenotypes of monozygotic twins with interstitial deletions of chromosome 1p

	Twin A	Twin B
Respiratory	Abnormal lobation of bilateral lungs (right lung with 1 fissure, left lung with no fissures)	Chronic lung disease, concern for airway malformation
Cardiovascular	Cardiomegaly, 2 midmuscular ventricular septal defects, high ostium secundum atrial septal defect, patent ductus arteriosus	Multiple small midanterior muscular ventricular septal defects, patent ductus arteriosus, pulmonary hypertension, ostium secundum ASD
Ear, nose, throat	Macroglossia, cleft palate, microretrognathia, low set ears, anteverted nostrils	Macroglossia, retroflexed epiglottis, retrodisplaced base of tongue, cleft of hard and soft palate, retrognathia, low set ears
Gastrointestinal	Meconium plugs, surgical necrotizing enterocolitis, hypoplastic spleen	Suspected Hirschsprung's disease, surgical necrotizing enterocolitis
Infectious disease	No definite infection diagnosed	*Enterobacter* pyelonephritis, MDR *E coli* pyelonephritis
Endocrinology	Hypoplastic adrenal glands, presumed cortisol deficiency	Low vitamin D, hypocalcemia, normal ACTH stimulation test
Nephrology	Bilateral simple cysts, salt‐wasting nephropathy, hypercalciuria, hematuria	Bilateral simple cysts, salt‐wasting nephropathy, nephrocalcinosis, hematuria
Ophthalmology	Right eye optic nerve and retinal coloboma	Pale, hypoplastic optic nerves
Neurology	Corpus callosum dysgenesis, ventriculomegaly, cortical or subcortical calcifications within the right frontal and parietal lobes, possible tethered spinal cord	Macrocephaly, ventriculomegaly, agenesis of the corpus callosum with subcortical punctate calcifications, diffusely abnormal gyral pattern, hypoplastic chiasm, tethered spinal cord, grade 3 IVH bilaterally and unilateral grade 4 IVH
Musculoskeletal	Abnormal skeletal proportions with smaller than expected crown‐rump and crown‐heel length, small hand length, asymmetric clefts within the right aspects of the S3 and S4 vertebral bodies, widely spaced nipples, bifid left hand 5th digit	Widely spaced nipples, shortened humeri, triphalangeal right 1st digit
Genetics	31.67 Mb de novo deletion 46,XX,del(1)(p22.2p32.2)	31.66 Mb de novo 46,XX,del(1)(p22.2p32.2)

### 2.1 Twin A

Twin A was vigorous at delivery and treated with continuous positive airway pressure (CPAP) for respiratory distress. Apgars were 8 and 9 at 1 and 5 minutes, respectively. Per the Fenton Premature Girl Growth Curve, birth weight was 1350 g (16th percentile), length was 39.6 cm (24th percentile), and occipital frontal circumference (OFC) was 28 cm (25th percentile). Her initial examination was notable for an anteriorly displaced but patent anus with friable tissue. There was concern for intestinal malrotation pattern on initial x‐ray. She received parenteral nutrition after birth and had ongoing feeding difficulties, first due to concern for bilious residuals and subsequently due to delayed passage of meconium. Twin A did not have a bowel movement until 15 days of life after a barium enema, which demonstrated meconium plugs. She tolerated gavage tube feedings of breast milk but developed necrotizing enterocolitis (NEC) at 3 weeks of age when feedings were fortified with human milk fortifier. She was clinically ill with NEC and progressed to modified Bell's stage IIIB NEC and required extensive bowel resection, which would have placed her at risk of short gut syndrome. Twin A required CPAP at birth for increased work of breathing. She was unable to wean off respiratory support and later required intubation during acute decompensation in the setting of evolving NEC. Her intubation was difficult due to macroglossia and micrognathia. Her echocardiogram showed at least two midmuscular ventricular septal defects (VSDs), a small‐moderate high ostium secundum atrial septal defect (ASD), and a large patent ductus arteriosus (PDA) with left to right flow. Blood culture drawn at birth due to preterm labor was negative. Serum toxoplasma IgM antibody and urine cytomegalovirus (CMV) were performed due to cortical calcifications and were negative. She was started on broad spectrum antibiotics for NEC based on institutional guidelines. She had an initial blood culture positive for coagulase‐negative Staphylococci aureus most likely due to contamination. A cranial ultrasound shortly after delivery was notable for corpus callosum dysgenesis, lateral ventriculomegaly measuring 8 mm bilaterally, and cortical or subcortical calcifications within the right frontal and parietal lobes. X‐rays of the spine were notable for asymmetric clefts within the 3rd and 4th sacral vertebral bodies, and sacral ultrasound was concerning for tethered spinal cord. Due to the callosal abnormality, hypopituitarism studies were obtained to evaluate for septo‐optic dysplasia and were all reassuring. The clinical team had planned an adrenocorticotropic hormone (ACTH) stimulation test when she was older and clinically stable, but this was not possible. Of note, when she was acutely ill with NEC, her random serum cortisol level was likely inappropriately low at 12.4 ug/dL. A renal ultrasound showed bilateral simple cysts. Right eye optic nerve and retinal colobomas were noted by ophthalmology. At 1 month of age, pediatric otolaryngology was consulted for a planned upsizing of her endotracheal tube due to significant leak. After her 3.0 endotracheal tube was removed, a new tube was unable to be placed due to her difficult airway. A code was called, and oral and nasal airways were attempted without improvement in bag mask ventilation. She required chest compressions and epinephrine until the pediatric otolaryngologist was able to place a tongue suture and reintubate. After this event, parents voiced that they did not want twin A to suffer and had concerns about her quality of life given her overall prognosis following bowel loss and concomitant diagnoses. Her parents chose to pursue palliative care, and she was compassionately extubated at their request. Parents consented to an autopsy, and specific findings are described in Table 1.

### 2.2 Twin B

Twin B was initially limp, cyanotic, and apneic at birth. She briefly required noninvasive positive pressure ventilation and then was transitioned to CPAP. Apgar scores were 3, 6, and 8 at 1, 5, and 10 minutes of life, respectively. Per the Fenton Premature Girl Growth Curve, birth weight was 1460 g (25th percentile), length was 38.5 cm (13th percentile), and OFC was 29.5 cm (45th percentile). Twin B's examination at birth was similar to her sister's, with anteriorly displaced anus and friable appearing tissue. She had a cleft palate. On her initial radiograph, the bowel gas pattern was concerning for intestinal malrotation. She also did not pass meconium until 16 days of age following barium enema; the pediatric surgery team was consulted, and she was managed with rectal irrigations. She was tolerating enteral feedings and rectal irrigations until 53 days of age when she developed medical NEC, which progressed to bowel perforation requiring laparotomy and repair but no bowel resection. After recovery and gradual advancement of enteral feedings, bloody stools intermittently recurred. A Meckel's scan was negative. Ultimately, she was treated for presumed milk protein allergy. Toward the end of her course, she developed feeding intolerance and was diagnosed with a colonic stricture. Twin B remained stable on CPAP until 2 days of age when she was intubated for increased respiratory distress. She was not able to be extubated to CPAP until 37 days. She later required intubation again during her bout of NEC, which was a difficult procedure due to retroflexed epiglottis and retrodisplaced base of tongue and required assistance by pediatric otolaryngology. She proved to have chronic lung disease and concern for upper airway malformation, requiring prolonged intubation. Once extubated, she was unable to maintain ventilation without bilevel positive airway pressure (BiPAP). Our pediatric pulmonologists were involved in her care, and tracheostomy was presented as the likely respiratory support she would require in order to survive outside of the hospital. Her postnatal echocardiogram revealed small muscular VSDs, ASD, and large PDA. Later, her echocardiogram showed pulmonary hypertension and right heart failure requiring inhaled nitric oxide, dopamine, and milrinone. Subsequent echocardiogram showed improvement in right ventricular function and medications were discontinued, though elevated pulmonary artery pressures persisted. Initial head and abdominal ultrasounds at 2 days revealed splenic and brain calcifications concerning for congenital infection. Serum toxoplasma IgM antibody and urine CMV were sent and negative. She was evaluated for sepsis at the time of birth given preterm labor. She subsequently received various courses of antibiotics in the setting of NEC, *Enterobacter* pyelonephritis, septic ileus, and multidrug‐resistant *Escherichia coli* pyelonephritis. Head ultrasound at 2 days of age revealed ventriculomegaly, agenesis of the corpus callosum, and cerebellar calcifications. Additionally, a spinal ultrasound was suggestive of tethered spinal cord. The head ultrasound was repeated at 4 days of age in the setting of clinical decompensation and drop in hemoglobin and showed large bilateral grade 3 intraventricular hemorrhage (IVH). Weekly monitoring ultrasound of her head revealed progressively worsening ventriculomegaly concerning for posthemorrhagic hydrocephalus as well as periventricular parenchymal hemorrhage (grade 4 IVH). Her hydrocephalus was managed with a ventriculo‐subgaleal shunt. Given her intracranial structural abnormalities, she was also evaluated for septo‐optic dysplasia but was not found to have hypopituitarism. A conference was held with the family and the subspecialty teams involved in twin B's care at 163 days of age to discuss her chronic lung disease, concern for upper airway anomaly, colonic stricture that would require an abdominal surgery, and neurologic prognosis. Multiple care plans were offered, including tracheostomy and gastrostomy tube, as well as redirection of goals of care to palliative care. After this meeting, parents made the difficult decision to redirect goals of care. Two days later, she was taken off BiPAP, provided medications and oxygen as needed for comfort, and passed away in her parents' arms. Her parents declined autopsy.

## 3 DISCUSSION

There have been ten published cases of de novo interstitial deletions of the short arm of chromosome 1 involving seven girls and three boys.^4^, ^7^, ^8^ A comparison of the manifestations of our two patients with previously reported cases is included in the Appendix S1. Varying degrees of cognitive impairment appear to be a common feature in addition to ocular defects, ear malformations, and other dysmorphic features, such as a broad nose tip, micrognathia, open mouth, short neck, clinodactyly, and other musculoskeletal abnormalities. The most severe phenotype reported, prior to our cases, was by Stockton et al^5^ with a postnatal karyotype revealing 46,XX,del(1)(p21p22.3). Clinical features included hypoplastic left heart syndrome, cleft lip and palate, absent thumb, and vertebral anomalies. That infant was discharged home with palliative care and survived 44 days. Maegawa et al, published the first report on a child with a fatty acid oxidation defect later also diagnosed with an interstitial deletion of chromosome 1p, which included the medium‐chain Acyl‐CoA dehydrogenase gene locus.^8^

To our knowledge, this is the first case report of monozygotic twins with a de novo 1p32.2‐p22.2 deletion (57, 627, 202‐89, 339, 298) as well as the first prenatally diagnosed case in the literature. There are several possible explanations for the more severe phenotypes observed in our patients, such as the extensive size of the deletion, the possibility of imprinting in the deleted region, and the confounding role of prematurity. We hypothesize that a vasculopathy, which has not been described in previous cases, may have predisposed our patients to surgical NEC, recurrent colonic bleeding, and intraventricular hemorrhage in twin B.

Given the rarity of reports, genetic counseling should address the wide spectrum of anomalies and outcomes associated with chromosome 1p interstitial deletions. It is possible that severe phenotypes similar to these twins will be identified more frequently as advancements in prenatal imaging and chromosomal testing with microarray analysis continue to become more widely available. However, spontaneous pregnancy loss or early neonatal death in the setting of large interstitial deletions could limit the identification of the full spectrum of clinical phenotypes. It is also unclear if there were any sequelae resulting from the mother's substance use during pregnancy that may have played a role in these twins' severe phenotype. Methamphetamine use has been associated with preterm birth and lower birth weight; however, other major adverse effects have not been described.^11^ Similarly, there are limited randomized controlled trials evaluating the impact of tetrahydrocannabinol (THC) use during pregnancy, and none suggest increased incidence of genetic disorders. Some observational studies, however, suggest that infants exposed to THC are at risk for lower birth weight and adverse long‐term behavioral and neurodevelopmental outcomes.^12^ We hope that future clinical descriptions and associated chromosomal mapping of interstitial deletions will help delineate the different roles of various regions of chromosome 1p and perhaps characterize a recognizable syndrome.

## CONFLICT OF INTEREST

The authors report no conflict of interest.

## AUTHOR CONTRIBUTIONS

Leah Yieh: made substantial contributions to the conception and design, performed the literature search, analyzed and interpreted data, drafted the manuscript, and revised it critically. Christina Ramo: performed the literature search, analyzed and interpreted data, drafted the manuscript, and revised it critically. Cori Feist: made substantial contributions to the manuscript draft, revised it critically for intellectual content, and helped obtain figures. Brian L. Shaffer: made substantial contributions to the conception and design and revised it critically for intellectual content. Amanda Kim: made substantial contributions to the conception and design, analyzed and interpreted data, and revised it critically for intellectual content.
