# Supplementary material for: Monozygotic dichorionic diamniotic twins with large interstitial deletion of chromosome 1p
Source: Clin Case Rep. 2019 Aug 6;7(9):1735–40. doi: 10.1002/ccr3.2358 (PMC6745365; doi:10.1002/ccr3.2358)
Supplement: Supplementary file 1 [file CCR3-7-1735-s001.docx]

Supplemental Table: Comparison of Manifestations of Our Patients with Previously Reported Cases of Interstitial Deletions of Chromosome 1p

|  | Twin A | Twin B | Bene et al. (1979) | Petersen et al. (1987) | Lai et al. (1991) | Lai et al. (1991) | Mattia et al. (1992) | Stockton et al. (1997) | Mircher et al. (2003) | Bisgaard et al. (2007) | Maegawa et al. (2008) |
| --- | --- | --- | --- | --- | --- | --- | --- | --- | --- | --- | --- |
| Genetics | 31.67 Mb *de novo* deletion  46,XX,del(1)(p22.2p32.2) | 31.66 Mb *de novo* 46,XX,del(1)(p22.2p32.2) | 46,XX,del(1)(p22p32), *de novo* | 46,XX,del(1)(p22-1  p31-2) | 46,XY,del(l) (p22.1p31.2) | 46,XY,del(l) (p22.3p31.3). | 46,XY,del(1)(p13p22.3) | 46,XX,del(1)(p21p22.3 | 46, XX,del (1)(p22p32), *de novo* | 11.9-14 Mb *de novo* deletion 46,XX,del(1)(p13.1p21.1) | 15.46 Mb 46,XX,del(1)(p22.2p31.1) |
| Respiratory | Abnormal lobation of bilateral lungs (right lung with 1 fissure, left lung with no fissures) | Chronic lung disease | Convex thorax; no respiratory insufficiency |  |  | “Apneic attacks” requiring hospitalization |  |  |  | Recurrent respiratory infections |  |
| Cardiology | Cardiomegaly, 2 mid-muscular ventricular septal defects, high ostium secundum atrial septal defect, patent ductus arteriosus | Multiple mid-anterior muscular ventricular septal defects, patent ductus  arteriosus, pulmonary hypertension, ostium-secundum atrial septal defect |  | Normal EKG |  |  | Normal echocardiogram, EKG | RVH on EKG, interrupted aortic arch, hypoplastic left ventricle, AV canal defect, primum and secumdum ASDs, VSD, interruption of hepatic segment of IVC |  |  | Moderate muscular VSD, PFO, mild pulmonary valve stenosis |
| Otolaryngology | Macroglossia, cleft palate with micro-retrognathia, low set ears, anteverted nostrils | Macroglossia, retroflexed epiglottis and prominent/retrodisplaced base of tongue, cleft of hard and soft palate, retrognathia, low set ears | Microretrognathia, small asymmetric ears, ridged tongue | Micrognathia, small primitive ears, large broad jaws, flat palate, poor dentition | Thick rounded helices, high palate, poor dentition |  | Low-set ears, micrognathia | Posteriorly-rotated, low-set ears, cleft lip and palate | Micrognathia, large, low-set ears, Bifid uvula | Low hairlines in front and back, low set ears, broad ear lobes, prominent crus helix of left ear, broad nasal bridge and tip, anteverted nares, prominent premaxillary region, open mouth with everted lower lip, high arched palate, space between upper incisors | Depressed nasal bridge, high arched palate, mild retromicrognathia, prominent antihelix bilaterally, raised vermillion borders |
| Gastroenterology | Meconium plugs,  surgical necrotizing enterocolitis, hypoplastic spleen | Suspected Hirschprung’s disease, surgical necrotizing enterocolitis |  |  |  |  | Gastroesophageal reflux, small bowel obstruction at four months requiring laparotomy |  | Gastroesophageal reflux; umbilical hernia |  |  |
| Infectious disease | No definite infection | Enterobacter pyelonephritis, MDR E. coli pyelonephritis |  |  |  | Died suddenly after mild upper respiratory tract infection |  |  |  |  |  |
| Endocrinology | Hypoplastic adrenal glands, presumed cortisol deficiency | Low vitamin D, hypocalcemia, | Short stature | Short stature, delayed puberty |  |  |  |  |  | Failure to thrive |  |
| Nephrology | Bilateral simple cysts, salt-wasting nephropathy, hypercalciuria, hematuria | Bilateral simple cysts, salt-wasting nephropathy, nephrocalcinosis, hematuria |  |  |  |  |  | small kidneys with a normal contour,  echogenicity, and position  Small kidneys, normal echogenicity, contour and position |  | Nor  Normal renal ultrasound |  |
| Ophthalmology | Right eye optic nerve and retinal coloboma | Pale, hypoplastic optic nerves |  | Bilateral microphthalmia with colobomas of the iris and choroid | Congenital absence of the left lens and a pupil abnormality with microphthalmia, hypertelorism |  |  |  | Hypopigmented retina | Coloboma of both irides and right optic disc, large right optic disc diameter with optic disc pit, concern for central vision impairment | Hypoplastic supra-orbital ridges |
| Neurology | Corpus callosum dysgenesis, ventriculomegaly, cortical or subcortical calcifications within the right frontal and parietal lobes, possible tethered spinal cord | Macrocephaly, ventriculomegaly, agenesis of the corpus callosum, subcortical punctate calcifications, diffusely abnormal gyral pattern, hypoplastic chiasm. Tethered spinal cord. IVH (grade III bilaterally, grade IV unilaterally) | Cognitive impairment | Microcephaly, cognitive impairment with speech never developed | Cognitive impairment | Hypertonia, developmental delay, abnormal discharges on EEG originating from left temporal region | Macrocephaly, hypotonia |  | Microcephaly, ventricular enlargement, increased pericerebral spaces, cognitive impairment | Epilepsy, severely delayed psychomotor and language development, diplegia, delayed myelination on MRI | Epileptiform discharges on EEG originating from bilateral temporal lobes, MRI with infarct in anterior limb of the left internal capsule (frontal lobe), prominent lateral ventricles and CSF fluid spaces suggestive of volume loss, global developmental delay, mild hypotonia of lower extremities |
| Musculoskeletal | Abnormal skeletal proportions with short crown-rump and crown-heel lengths, small hand length, asymmetric clefts in S3 and S4 vertebral bodies, widely spaced nipples, bifid left hand 5th digit | Widely spaced nipples, shortened humeri, tri-phalangeal right 1^st^ digit | Short neck, clinodactyly of 5^th^ finger sand toes, enlarged space between 1^st^ and 2^nd^ toes, genu valgum | Short, neck, clinodactyly of left fifth finger, proximal implantation of the thumbs, mild dysplasia of left iliac bone | Clinodactyly of the 5^th^ fingers, bilateral single palmar creases, short, proximally implanted thumbs, joint laxity | Long fingers, joint laxity, proximally implanted thumbs | Joint laxity, high arched right foot, | Short neck, inverted nipples, left thumb absent, long thin fingers, bilateral radial deviation of wrists, radial hypoplasia, rib anomalies and butterfly vertebrae | Short neck, joint laxity, tapered fingers | Narrow shoulders, webbed neck, valgus feet, groove between 1^st^ and 2^nd^ toes, 2-3 syndactyly on right foot | Prominent forehead, flattened occiput, pectus excavatum, inverted nipples |
| Metabolic |  |  |  |  |  |  |  |  |  |  | Medium-chain Acyl-CoA dehydrogenase deficiency |
| Outcome | Death at 1 month after re-direction of care | Death at 5 months after re-direction of care | Living at time of publication | Living at time of publication | Living at time of publication | Death at 7 months | Living at time of publication | Death at 44 days of life after re-direction of care | Living at time of publication | Living at time of publication | Living at time of publication |
